# Transmission Chains of Extended-Spectrum Beta-Lactamase-Producing Enterobacteriaceae at the Companion Animal Veterinary Clinic–Household Interface

**DOI:** 10.3390/antibiotics10020171

**Published:** 2021-02-09

**Authors:** Kira Schmitt, Stefan P. Kuster, Katrin Zurfluh, Rahel S. Jud, Jane E. Sykes, Roger Stephan, Barbara Willi

**Affiliations:** 1 Institute for Food Safety and Hygiene, University of Zurich, Winterthurerstrasse 272, CH-8057 Zurich, Switzerland; kira.schmitt@uzh.ch (K.S.); katrin.zurfluh@uzh.ch (K.Z.); 2 Graduate School for Cellular and Biomedical Sciences, University of Bern, Mittelstrasse 43, CH-3012 Bern, Switzerland; 3 Division of Infectious Diseases and Hospital Epidemiology, University Hospital Zurich, University of Zurich, Rämistrasse 100, CH-8091 Zurich, Switzerland; stefan.kuster@usz.ch; 4 Division of Critical Care Medicine, Department of Small Animals, University of Zurich, Winterthurerstrasse 260, CH-8057 Zurich, Switzerland; rahel.jud@uzh.ch; 5 Department of Medicine & Epidemiology, University of California-Davis, 2108 Tupper Hall, Davis, CA 95618, USA; jesykes@ucdavis.edu; 6 Clinic for Small Animal Internal Medicine, University of Zurich, Winterthurerstrasse 260, CH-8057 Zurich, Switzerland; bwilli@vetclinics.uzh.ch

**Keywords:** antimicrobial resistance, multidrug resistance, canine, feline, ESBL, home, hospital, high-risk clone *Klebsiella pneumoniae* ST307

## Abstract

Extended-spectrum beta-lactamase-producing Enterobacteriaceae (ESBL-E) among animals and humans are a public health threat. This study analyzed the occurrence of ESBL-E in a high-risk environment in a companion animal clinic and two animal patients’ households. In an intensive care unit (ICU), rectal swabs from 74 dogs and cats, 74 hand swabs from staff and 298 swabs from surfaces were analyzed for ESBL-E. Seventeen hospitalized patients (23%) and ten (3%) surfaces in the ICU tested ESBL-E positive. Transmission chains for *Klebsiella pneumoniae* ST307 *bla*_CTX-M-15_ and *Escherichia coli* ST38 *bla*_CTX-M-14_, ST88 *bla*_CTX-M-14_ and ST224 *bla*_CTX-M-1_ were observed over extended periods of time (14 to 30 days) with similar strains isolated from patients and the clinical environment. After discharge, two colonized dogs (dogs 7 and 12) and their household contacts were resampled. Dog 7 tested repeatedly positive for 77 days, dog 12 tested negative; six (24%) surfaces in the household of the persistently colonized dog tested ESBL-E positive. The owner of dog 7 and one of the owners of dog 12 were colonized. Based on whole genome sequencing, isolates from the owners, their dogs and other ICU patients belonged to the same clusters, highlighting the public health importance of ESBL-E in companion animal clinics.

## 1. Introduction

Antimicrobial resistance in companion animals is of public health importance because of the close contact between pets and their owners, which can facilitate the transmission of resistant bacteria [1,2,3,4,5,6,7,8,9]. The trend towards intensive medical care of dogs and cats fosters hospitalization and nosocomial infections [10,11,12,13] and has led to a growing number of geriatric and immunosuppressed animal patients that are highly susceptible to infections, including those with antimicrobial resistant microorganisms (ARM). Antimicrobial use, which is discussed as one of the main drivers of resistance development, is common in companion animal medicine, including the use of highest priority critically important antimicrobials and even antibiotics of last resort, such as carbapenems, are administered in some instances [14,15,16,17,18,19,20,21,22,23,24].

The spread of ARM, such as extended-spectrum beta-lactamase-producing Enterobacteriaceae (ESBL-E), challenges human and veterinary healthcare settings worldwide and poses a public health threat [25]. In addition to their plasmid-mediated resistance to penicillins and cephalosporins, ESBL-E are often resistant to antibiotics such as fluoroquinolones, aminoglycosides, and sulfamethoxazole/trimethoprim [26]. Previous hospitalization, a raw food diet, elderly age, urinary or intra-abdominal infections, hepatic cirrhosis, residence in overcrowded household districts and antimicrobial therapy are known risk factors for ESBL-E colonization of dogs and cats [27,28,29]. In a recently published study, 21.4% of dogs and cats carried ESBL-E on admission to veterinary hospitals, whereas 53.7% were colonized after 72 h of hospitalization [27]. This points towards an important role of companion animal clinics in the transmission of ESBL-E [1,10,11,12,13,30]. However, the transmission chains for ESBL-E within veterinary hospitals, especially in high-risk settings such as intensive care units (ICUs), has not yet been resolved. Additionally, the impact of colonized patients for ESBL-E dissemination in the households after discharge is unclear.

The close contact of companion animals in the household to their owners is thought to be a risk factor for ARM transmission to owners. In households in which humans carry ESBL-E, identical strains were detected in dogs from the same households [28,31]. Furthermore, a study from human medicine documented that transmission rates of ESBL-E between humans in household settings outnumbered transmission rates within the hospital, and transmission rates of 23% and 25% for ESBL-producing *Escherichia coli* (*E. coli*) and *Klebsiella pneumoniae* (*K. pneumoniae*), respectively, were documented within the households [32]. This indicates that household transmission between humans can play a substantial role in the spread of ESBLE-E, but data regarding transmission between companion animals and humans in households is limited. Furthermore, the contamination of the household environment with ESBL-E by colonized pets has not yet been investigated.

The aims of this study thus were to analyze transmission chains of ESBL-E over a 45-day period in an intensive care unit, a high-risk environment in a companion animal clinic, and to investigate ESBL-E dissemination by colonized patients to household contacts and the environment in two households after discharge.

## 2. Results

### 2.1. ESBL-E in the Intensive Care Unit

**Figure 1:**
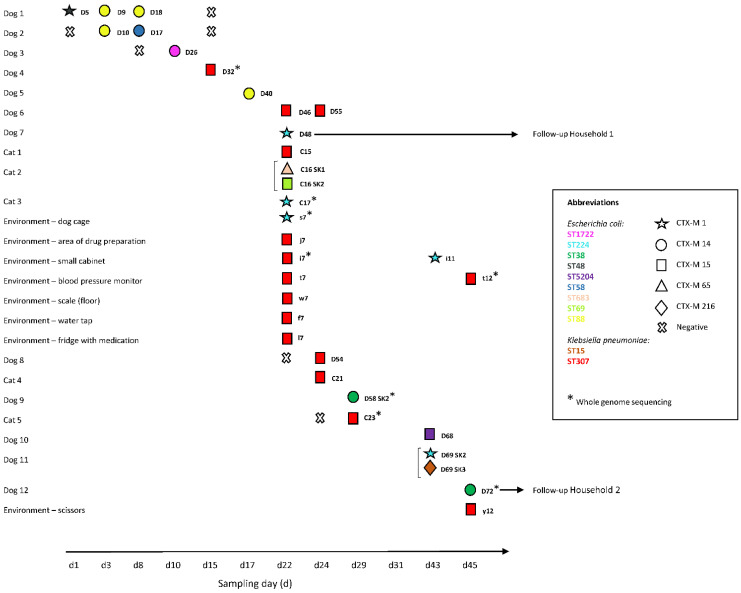
Timeline of extended-spectrum beta-lactamase-producing Enterobacteriaceae (ESBL-E) isolated from dogs, cats and the clinical environment. D, dog; C, cat; d, sampling day; s, dog cage; j, area of drug preparation; i, small cabinet; t, blood pressure monitor; w, scale (floor); f, water tap; l, fridge with medication; y, scissors; SK, subculture. Each horizontal line refers to a specimen obtained from the same animal or environmental surface over time. The brackets at the left side of the strain ID indicate subcultures of the same specimen. Negative test results are only shown for animals or environmental surfaces that had tested positive for ESBL-E at a certain point in time.

A total of 91 rectal swab specimens from 49 dogs and 25 cats hospitalized in the ICU, and 298 specimens from 25 predefined high–touch surfaces and from 74 hands from healthcare workers in the ICU were collected at regular intervals on 12 sampling days over a 45-day period. ESBL-E (*E. coli* and *K. pneumoniae*) were isolated from 12 (24%) dogs and 5 (20%) cats (Figure 1, Table S1) and from 3% of the high-touch surfaces (range: 0–28% per sampling day; positive specimens: dog cage, area of drug preparation, small cabinet, blood pressure monitor scale (floor), water tap, fridge with medication, scissors). None of the hand swabs tested positive for ESBL-E.

**Figure 2:**
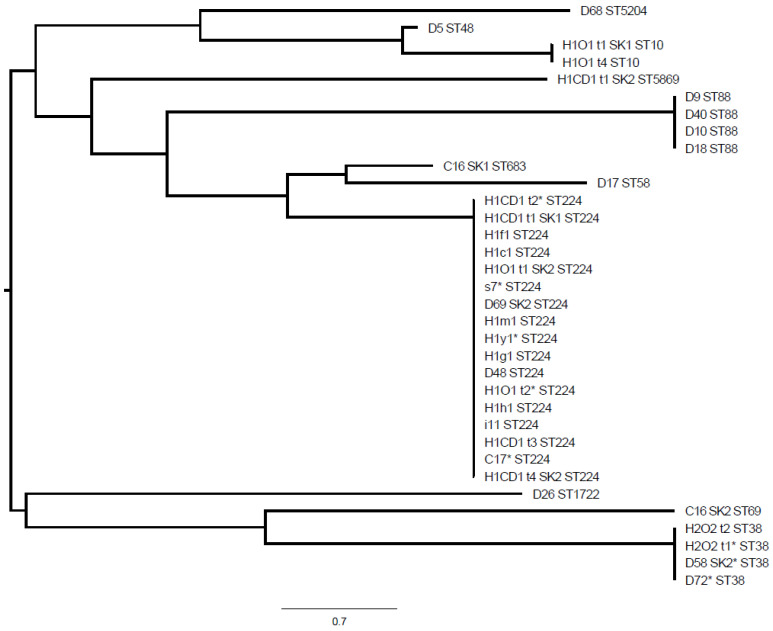
Multi-locus sequence typing-distance based phylogenetic tree for extended-spectrum beta-lactamase-producing *Escherichia coli* isolates. D, dog; C, cat; CD, colonized dog; O, owner; lower case letter, environment; H1, household 1; H2, household 2; D68, dog 10; D5, dog 1; H1O1 t1 SK1, owner household 1; H1O1 t4, owner household 1; H1CD1 t1 SK2 dog household 1; D9, dog 1; D40, dog 5; D10, dog 2; D18, dog 1; C16 SK1, cat 2; D17, dog 2; H1CD1 t2, dog household 1; H1CD1 t1 SK1, dog household 1; H1f1, dog’s sleeping basket (living room); H1c1, water bowl; H1O1 t1 SK2, owner household 1; s7, dog cage; D69 SK2, dog 11; H1m1, carpet; h1y1, kitchen sponge; H1g1, dog’s blanket on terrace; D48, dog 7; H1O1 t2, owner household 1; H1h1, dog’s sleeping basket (bedroom); i11, small cabinet; H1CD1 t3, dog household 1; C17, cat 3; H1CD1 t4 SK2, dog household 1; D26, dog 3; C16 SK2, cat 2; H2O2 t2, owner household 2; H2O2 t1, owner household 2; D58 SK2, dog 9; D72, dog 12; *, whole genome sequencing conducted.

**Figure 3:**
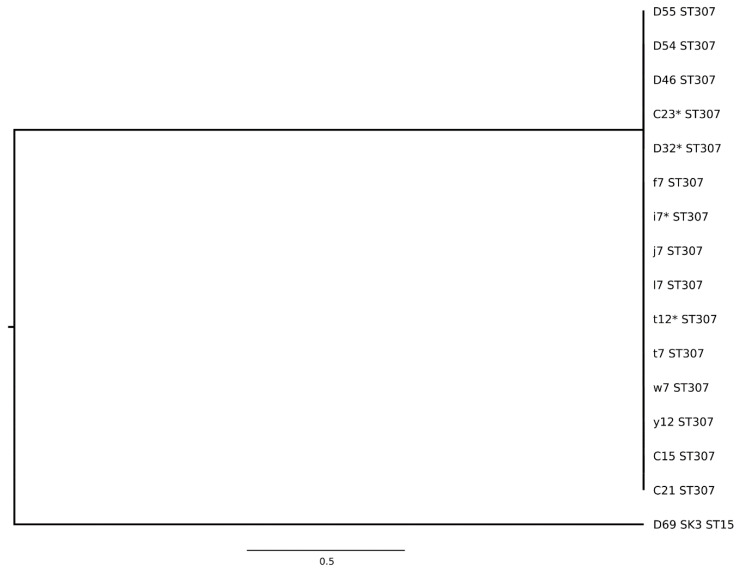
Multi-locus sequence typing-distance based phylogenetic tree for extended-spectrum beta-lactamase-producing *Klebsiella pneumoniae* isolates. D, dog; C, cat; O, owner; lower case letter, environment; D55, dog 6; D54, dog 8; D46, dog 6; C23, cat 5; D32, dog 4; f, water tap; i, small cabinet; j, area of drug preparation; l, fridge with medication; t, blood pressure monitor; w, scale (floor); y, scissors; C15, cat 1; C21, cat 4; D69 SK3, dog 11; *, whole genome sequencing conducted.

ESBL-E genes detected in the clinic included *bla*_CTX-M-1_, *bla*_CTX-M-14_, *bla*_CTX-M-15_, *bla*_CTX-M-65_, and *bla*_CTX-M-216_; *bla*_CTX-M-15_ was most common and detected in 8 of 10 (80%) environment- and 10 of 23 (43%) patient-derived ESBL-E positive specimens. Additionally, broad spectrum beta-lactamase genes *bla*_SHV-1_ and *bla*_TEM-1_ were detected amongst these isolates (Table S1). Among the *E. coli* isolates, nine different sequence types were identified (Figure 1, Table S1). Among the *K. pneumoniae* isolates, ST15 and ST307 were found. The phylogenetic relationship for all human-animal-environmental strains is shown in Figure 2 and Figure 3. *K. pneumoniae* ST307 *bla*_CTX-M-15_ predominated in the ICU, particularly on day 22 where ESBL-E contamination of the ICU was most extensive (Figure 1, Table S1). On this day, 7 (28%) environmental specimens tested positive for ESBL-E and six of these isolates belonged to *K. pneumoniae* ST307 *bla*_CTX-M-15_.

Transmission chains for several closely related ESBL-E isolates were detected within the ICU over extended periods of time. *K. pneumoniae* ST307 *bla*_CTX-M-15_ was isolated for the first time from dog 4 on day 15 and thereafter from different hospitalized patients (dogs 6 and 8; cats 1, 4 and 5; days 22–29) and environmental surfaces (days 22 and 45, Figure 1), which indicates an ongoing transmission chain for this strain. Some of these isolates (dog 4, day 15; cat 5, day 29; environmental specimens, days 22 and 45) were characterized by whole genome sequencing (WGS) and core genome multi-locus sequence typing (cgMLST) analysis which revealed that all selected isolates belonged to the same cluster.

Additionally, three specific *E. coli* strains (ST88 *bla*_CTX-M-14_, ST224 *bla*_CTX-M-1_, ST38 *bla*_CTX-M-14_) were isolated on various sampling occasions in the ICU. *E. coli* ST88 *bla*_CTX-M-14_ was detected in dog 1 (days 3 and 8) and dog 2 (day 3) and was thereafter isolated from dog 5 on day 17. *E. coli* ST224 *bla*_CTX-M-1_ first occurred in dog 7, cat 3 and the environment on day 22, and was detected again 21 days later (day 43) in dog 11 and in the clinical environment. Lastly, *E. coli* ST58 *bla*_CTX-M-14_ was isolated on a first occasion from in dog 9 on day 29 and again from dog 12 on day 45.

### 2.2. ESBL-E in the Households

**Figure 4:**
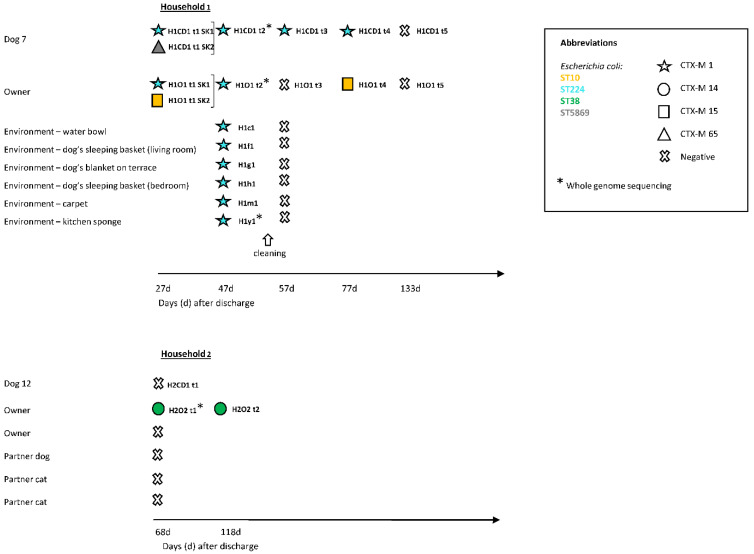
Timeline of extended spectrum beta-lactamase producing Enterobacteriaceae (ESBL-E) isolated from the dogs, cats and owners in household 1 and 2 and the household environment. CD, colonized dog; O, owner; d, days after discharge; H1, household 1; H2, household 2; H1c1, water bowl; H1f1, dog’s sleeping basket (living room); H1g1, dog’s blanket on terrace; H1h1, dog’s sleeping basket (bedroom); H1m1, carpet; H1y1, kitchen sponge; SK, subculture. Each horizontal line refers to a specimen obtained from the same animal, owner or environmental surface over time (12, t2, t3, t4, t5). The brackets at the right side of the strain ID indicate subcultures of the same specimen. Negative test results are only shown for animals, environmental surfaces or owners that had tested positive for ESBL-E at a certain time point.

Two colonized dogs (dog 7, household 1; dog 12, household 2) were resampled at home after discharge from the clinic, together with the household contacts and the household environment (Figure 4). Household 1 contained dog 7 and the owner. Dog 7 and the owner were found to be persistently and intermittently colonized with ESBL-E, respectively, between days 27 and 77 after the dog’s discharge from the clinic, and both tested negative on day 133. *E. coli* ST224 *bla*_CTX-M-1_ and ST5869 *bla*_CTX-M-56_ were detected in the dog, and *E. coli* ST224 *bla*_CTX-M-1_ and ST10 *bla*_CTX-M-15_ in the owner. *E. coli* ST224 *bla*_CTX-M-1_ had already been isolated from dog 7 during hospitalization (and from other ICU patients and the ICU environment, see above) and was found in dog 7 on repeated samplings until day 77 after discharge and in the owner until day 47 after the dog’s discharge (Figure 4). Isolates of *E. coli* ST224 *bla*_CTX-M-1_ from dog 7 (day 47 after discharge), its owner (day 47 after discharge) and clinic-derived specimens (cat 3, day 22; clinical environment, day 22) were subject to WGS and cgMLST analysis and confirmed to belong to the same cluster.

Environmental contamination with ESBL-E was detectable in 6 (24%) specimens in household 1 (at day 47 after discharge) and all isolates belonged to *E. coli* ST224 *bla*_CTX-M-1_. Areas in close contact with the dog, such as the carpet, the dog’s water bowl, the dog’s sleeping basket in the living room, the dog’s blanket on the terrace and the dog’s sleeping basket, but also the kitchen sponge, were contaminated, whereas areas primarily in contact with the owner tested negative. The relatedness of the isolate deriving from the kitchen sponge to the other *Escherichia coli* ST224 *bla*_CTX-M-1_ was confirmed through WGS and cgMLST. Immediately after thorough cleaning with a commercially available household cleaning product, none of the environmental specimens taken tested positive (day 57), while the dog, but not the owner, remained consistently positive until day 77 (Figure 4).

Household 2 contained two people, two cats, the colonized dog 12 and another dog (Figure 4). At the time of retesting (68 days after discharge), one of the two owners in household 2 was colonized with *E. coli* ST38 *bla*_CTX-M-14_ while both dogs, the cats and the other owner tested negative (Figure 4). The owner tested again positive for this strain in the second sample collected 118 days after the dog’s discharge. *E. coli* ST38 *bla*_CTX-M-14_ had originally also been isolated from dog 12 of this household (on day 45 during hospitalization), and in another dog (dog 9, day 29) from the ICU 16 days before dog 12 was sampled. WGS and cgMLST analysis confirmed that the isolates of dogs 9 and 12 and of the owner of dog 12 (68 days after discharge) belonged to the same cluster. Environmental contamination with ESBL-E was undetectable in this household (at day 68 after discharge) where only one owner, but not the dogs, was colonized with ESBL-E.

Hygiene standards were assessed in both households using a questionnaire (Table S2). Overall, hygiene behavior did not clearly differ between the households. Owners of both households indicated that they “regularly” used hand sanitizer and antibacterial soap, none of the owners fed their dogs a raw food diet but both owners had contact to the human health care system (the owner living in household 1 worked as a surgical cosmetician, the owner in household 2 worked as a care professional in a nursing home). Owners from both households indicated that kitchen towels were not changed daily and that no separate chopping boards were used for meat and food of nonanimal origin.

### 2.3. Resistance Profiles of ESBL-E

Resistance profiles were determined for all ESBL-E isolates collected in this study. The strains shared by the owner and the colonized dogs in households 1 and 2 showed resistance to ampicillin, cephazolin, cefotaxime, cephepime, nalidixic acid, ciprofloxacin, sulfamethoxazole-trimethoprim and streptomycin (Table S1). The *E. coli* strains in household 1 were additionally resistant to kanamycin, gentamicin and tetracycline, whereas the *E. coli* strains in household 2 and from dogs 9 and 12 were also resistant to azithromycin.

## 3. Discussion

The present study documents transmission chains for several ESBL-producing *E. coli* and *K. pneumoniae* strains in a high-risk setting of a companion animal clinic. Within the limited time of observation (45 days), transmission chains for one *K. pneumoniae* and three *E. coli* strains were documented, and the isolates included high-risk human pathogenic clones such as *K. pneumoniae* ST307 which has been previously associated with carbapenem and ESBL resistance [33,34,35,36]. This strain was repeatedly detected in ICU patients and the clinical environmental over a period of 30 days, indicating an ongoing outbreak situation. Our results underline that ICU settings in companion animal clinics could significantly contribute to the spread of ESBL-E and high-risk human pathogenic clones.

Worryingly, the study also supports a direct transfer of ESBL-producing *E. coli* strains from ICU patients to companion animal owners (or vice versa). A recent study found that only 12% of the owners in households with a colonized dog were ESBL-E carriers. Additionally, a match in the core genome between the owner and the dog specimen was only found in 5% of the exposed households [28]. A previous study found that dog ownership was not a risk factor for ESBL-E carriage [29], however, dogs are often colonized with ESBL-E after hospitalization [27]. *E. coli* ST38 *bla*_CTX-M 14_ originally detected in two ICU patients was isolated from the owner of one of these animal patients after the dog’s discharge. Interestingly, the colonization of the owner was found at a time when the dog tested negative for this strain, and colonization persisted for at least 50 days. WGS and cgMLST confirmed the very close relationship of the isolates of the owner and the ICU patients. Of note, the colonization of the owner was not associated with environmental contamination with ESBL-E in this household. In the second household investigated in this study, closely related *E. coli* ST224 *bla*_CTX-M 1_ isolates were detected in the owner and its dog after discharge over extended periods of time, and considerable environmental household contamination occurred with this strain (24% positive environmental surfaces). The dog remained colonized with this *E. coli* strain for 77 days after discharge. In this household, it was unclear whether the dog introduced this *E. coli* strain into the ICU and caused a transmission chain to three other ICU patients, or whether colonization first occurred during hospitalization and resulted in a transfer of the isolate into the household. Again, WGS and cgMLST confirmed the very close relationship of the clinic- and owner-derived isolates. The results underline that ESBL-E transmission chains can be frequent in ICU settings in companion animal clinics and pose a risk for both, the animal owners and for other ICU patients. Interruption of these transmission chains by comprehensive infection prevention and control (IPC) concepts including stringent adherence to hand hygiene are thus of public health importance and should be urgently promoted. So far, the implementation IPC concepts are, in contrast to human hospitals, not mandatory for veterinary clinics in Switzerland.

Households have been previously described as a potential reservoir for ESBL-E [37,38]. Environmental contamination with ESBL-E in the household was extensive in household 1 with the persistently colonized dog but was undetectable in household 2 where only the owner was colonized, although hygiene habits seemed to be comparable in the two investigated households. Both owners indicated not using separate chopping boards for meat and food of nonanimal origin and not changing kitchen towels daily, which could both be an important source of transmission of ESBL-E [29,39]. Furthermore, ESBL-E were primarily detected on surfaces in household 1 that were in close contact with the persistently colonized dog. This could support the hypothesis that colonized companion animals contribute more to household contamination with ESBL-E than colonized humans. This is also supported by the fact that in household 1, *E. coli* ST224 *bla*_CTX-M 1_ was the only detected strain in the dog during hospitalization and for 77 days after discharge, in contrast to the owner, who was colonized with an additional strain, and only *E. coli* ST224 *bla*_CTX-M 1_ was detected in the household environment. Such long carriage periods could additionally contribute to the risk of spreading of ESBL-E in the household environment [28]. Of note, environmental contamination in household 1 for ESBL-E was much higher than at most of the sampling days in the ICU in the investigated clinic (3%, range: 0–24%), and higher than recently reported for environmental samples collected in seven companion animal clinics and practices in Switzerland (0–2% of the environmental specimens were ESBL-E positive) [40]. Although only two households were investigated in this study, our results are alarming and highlight the need to develop evidence-based recommendations for the handling of ESBL-E colonized animals in the household environment.

Overall, *bla*_CTX-M-15_, a highly prevalent ESBL gene in both humans and companion animals, predominated in the clinical samples [41,42,43,44]. The previously described emergence of *bla*_CTX-M-1_ and *bla*_CTX-M-14_ in dogs and cats in Switzerland was also evident in this study [45]. ST307 has been previously described in a dog with a urinary tract infection from Brazil [46]. Additionally, *E. coli* ST38 *bla*_CTX-M-14_, ST88 *bla*_CTX-M-14_ and ST224 *bla*_CTX-M-1_ reoccurred on different sampling occasions indicating additional minor outbreaks. ST224, which was also isolated from the owner in this study, has been frequently reported in companion animals [47,48,49]. ST88 is common among both humans and animals and globally distributed [50]. Both ST88 and ST38 belong to global extraintestinal pathogenic *E. coli* lineages and companion animals have been documented as a possible reservoir [51,52]. Furthermore, ST15, an epidemic and international human-related *K. pneumoniae* strain, was isolated from one dog in this study. *K. pneumoniae* ST15 and *E. coli* ST10 and ST58 strains have been previously isolated from clinical specimens of companion animal patients from the same veterinary clinic in an unrelated study [45].

Data on environmental contamination by ESBL-E in veterinary facilities are scarce. A recent study reported a prevalence of ESBL-E on high-touch surfaces ranging from 0–2% across seven Swiss veterinary clinics and practices [40] and areas with high patient traffic and utensils were most contaminated with ARM [40]. In this study, environmental contamination with ESBL-E in a high-risk setting was detected in 3% of the high-touch surfaces, but contamination varied considerably between the sampling days: seven of 10 isolates were found on sampling day 22 and six of these seven specimens yielded *K. pneumoniae* ST310 *bla*_CTX-M-15_.

ESBL-E was not isolated from any of the hand swabs in this study although hands are regarded as one of the main vectors for ARM transmission. Considering the high number of hand-animal contacts that take place during the daily work of healthcare workers, the microbiological analyses of the swabs represent only a snapshot and cannot fully mirror the transmission events in these settings. Previous studies have reported drug-resistant Enterobacteriaceae on the hands of veterinary staff [53] and nosocomial pathogens have been isolated from the hands of healthcare workers [54].

The present study has some limitations. The ICU of only one veterinary clinic was investigated in this study. An extrapolation of our results to ICU settings of other veterinary clinics is thus not possible. Of note, the companion animal clinic included in this study showed the lowest environmental ARM contamination and the highest IPC standards among three large referral clinics in Switzerland in a recent study, suggesting that the frequency of ESBL-E transmission chains observed in this study might not be overestimated [40]. However, the present study also showed considerable variations in the ESBL-E detection between sampling days. Furthermore, the present study investigated only two households, and documentation of a colonized companion animal was only available in one of the households. Furthermore, environmental sampling in the household differed regarding the time after discharge. Future studies should thus further elucidate the ESBL-E transmission chains between companion animals and owners in household settings.

## 4. Materials and Methods

### 4.1. Ethics

All methods were carried out in accordance with relevant guidelines and regulations. In accordance with local legislation, ethical approval was sought from the Swiss Ethics Committees on research involving humans (2019-00768). Informed consent was obtained from all participants. Ethical approval for the collection of rectal swab specimens from the dogs and cats was received from the local Veterinary Office (ZH028/19). All owners of the dogs and cats gave informed consent.

### 4.2. Study Set-Up

#### 4.2.1. Specimen Collection

Single cotton swabs were used for the collection of specimens. Rectal swab specimens from all dogs and cats that were examined by the intensive care unit (ICU) of a veterinary tertiary care facility between June 2019 and August 2019 were collected by the first author of this study after informed owner consent. Sampling intervals were kept constant throughout the study period (Figure 1). Swabs from a modified previously published list of high-touch surfaces (Table S3) were collected in the ICU during the same time period [40]. During the same time points, hand swabs of the dominant hand from veterinary staff (i.e., veterinarians, nurses and students) working in the ICU were collected before and after animal patient contact, regardless as to whether gloves were worn. If gloves were worn, the hand swab was taken from the glove.

Two households were followed-up and were asked to send stool specimens of the colonized animal patient and its household contacts (owner, dogs and cats living in the same household) at different time points (Figure 4) and specimens from twenty-five surfaces with high human and animal contact in the household (Table S4) were collected. Furthermore, owners were asked to fill out a questionnaire on household hygiene (Table S2) [55,56,57]. There was no compensation for participating in the study.

#### 4.2.2. Microbiological Analysis

Specimens from dogs, cats and owners of two colonized dogs, hand swabs and swabs of high-touch surfaces from the clinic and the household environment were analyzed for the presence of ESBL-E.

All swabs were immediately enriched in 10 mL peptone water (BioRad, Hercules, CA, USA), followed by selective enrichment in Enterobacteriaceae enrichment broth (Oxoid, Hampshire, UK). ESBL-E were screened by using the chromogenic medium Brilliance™ ESBL Agar (Oxoid, Hampshire, UK), according to the manufacturer’s instructions. Colonies were picked from the selective media based on phenotype and species identification was conducted by using matrix-assisted laser desorption/ionization time-of-flight mass spectrometry (MALDI-TOF–MS, Bruker Daltronics, Bremen, Germany). Polymerase chain reaction (PCR) assays for the presence of genes encoding *bla*_CTX-M_ groups, *bla*_SHV_ and *bla*_TEM_ were conducted on Enterobacteriaceae isolates as previously described [45,58,59,60].

#### 4.2.3. Antimicrobial Susceptibility Testing

Antimicrobial susceptibility testing was performed for Enterobacteriaceae in accordance with the Clinical and Laboratory Standards Institute (CLSI) performance standards [61] using the disk-diffusion method on Mueller Hinton plates (Oxoid, Hampshire, UK) and the antibiotics ampicillin (AM), amoxicillin with clavulanic acid (AMC), azithromycin (AZM), cefazolin (CZ), cefepime (FEP), cefotaxime (CTX), chloramphenicol (C), ciprofloxacin (CIP), fosfomycin (FOS), gentamicin (G), kanamycin (K), nalidixic acid (NA), nitrofurantoin (F/M), streptomycin (S), sulfamethoxazole trimethoprim (SXT) and tetracycline (TE) (Becton Dickinson, Allschwil, Switzerland). Results were interpreted according to CLSI standards [61]. For azithromycin, an inhibition zone of ≤12 mm was interpreted as resistant.

#### 4.2.4. MLST

Sequence type determination of the identified Enterobacteriaceae isolates, i.e., *E. coli* and *K. pneumoniae*, was carried out as published previously [62,63]. Sequence analysis was conducted using Ridom^TM^ SeqSphere+ (Ridom© GmbH, Münster, Germany).

#### 4.2.5. WGS

Whole genome sequencing was performed according to procedures previously described on selected isolates from colonized dogs and cats, owners and clinical and household environment based on MLST results, genes encoding *bla*_CTX-M_ groups, *bla*_SHV_ and *bla*_TEM_ and antimicrobial susceptibility testing [64]. Briefly, the isolates were grown overnight on sheep blood agar at 37 °C prior to genomic DNA isolation using the DNA blood and tissue kit (Qiagen, Hombrechtikon, Switzerland). A Nextera DNA Flex Sample Preparation Kit (Illumina, San Diego, CA, USA) was used to prepare the DNA, which produces transposome-based libraries that were sequenced on an Illumina MiniSeq Sequencer (Illumina, San Diego, CA, USA). Reads were checked for quality using the software package FastQC 0.11.7 (Babraham Bioinformatics, Cambridge, UK). Both Illumina-reads files passed the standard quality checks of FastQC, with the exception of the module “Per Base Sequence Content”, which returned a failure. Such failure is common for transposome-based libraries and was therefore ignored and reads were assembled using the Spades 3.0 based software Shovill 1.0.4, using default settings. The assembly was filtered, retaining contigs > 500 bp.

## 5. Conclusions

In conclusion, the present study documents transmission chains for the human pathogenic *K. pneumoniae* ST307 strain and three ESBL-producing *E. coli* strains in an ICU of a veterinary clinic over a 45-day observation period. The study strongly suggests the transfer of ESBL-producing *E. coli* strains from the ICU setting to the patients’ households and pet owners, and vice versa, with extended periods of ESBL-E colonization in the animals and owners. Contamination of the household environment in the case of a persistently colonized dog was extensive and might outweigh contamination by colonized humans. The study highlights the risk of veterinary clinics in the spread of ARM and the need to further investigate transmission of ARM in companion animal households in order to develop evidence-based recommendations on hygiene measures in these settings.

## Data Availability

Sequence and annotation data of the genomes have been deposited in GenBank under accession numbers JAANCW000000000 (C17), JAANCY000000000 (C23), JAANCV000000000 (D32), JAANCU000000000 (D72), JAANCQ000000000 (H1CD1_t2), JAANCS000000000 (H1O1_t2), JAANCR000000000 (H1y1), JAANCP000000000 (H2O2_t1), JAANCZ000000000 (i7), JAANCT000000000 (s7), JAANCX000000000 (t12), JADANJ000000000 (D58 SK2).

## Supplementary Materials

The following are available online at https://www.mdpi.com/2079-6382/10/2/171/s1, Table S1: Extended-spectrum beta-lactamase-producing Enterobacteriaceae (ESBL-E) isolated from dogs, cats, clinical and household environment and owners of colonized pets, Table S2: Questionnaire for the owners of colonized pets, Table S3: List of high-touch surfaces in the veterinary clinic, Table S4: List of high-touch surfaces in the households of colonized pets.
