# Supplementary material for: Transmission Chains of Extended-Spectrum Beta-Lactamase-Producing Enterobacteriaceae at the Companion Animal Veterinary Clinic–Household Interface
Source: Antibiotics (Basel). 2021 Feb 9;10(2):171. doi: 10.3390/antibiotics10020171 (PMC7914568; doi:10.3390/antibiotics10020171)
Supplement: Supplementary file 1 [file antibiotics-10-00171-s001.pdf]

Figures for:

Table S1: Extended spectrum beta-lactamase producing Enterobacteriaceae (ESBL-E) isolated from dogs, cats, clinical and household environment and owners of colonized pets

			Sample type							Species			*bla* gene							Zone diameters [mm]															
Strain ID		Sampling day	Clinic	Dog	Cat	Environment	Household 1	Household 2	Owner	*E. coli*	*K. pneumoniae*	MLST (CC)	CTX-M-1	CTX-M-14	CTX-M-15	CTX-M-65	CTX-M-216	SHV-1	TEM-1	AM	CZ	CTX	AMC	FEP	NA	CIP	SXT	FOS	AZM	F/M	S	K	GM	C	TE
D5	D5_t1	1	■	■						■		ST48 (CC10)	■						■	6	6	17	16	22	24	29	6	32	14	28	6	6	9	24	6
D9	D5_t2	3	■	■						■		ST88 (CC23)		■						6	6	15	20	20	8	25	25	33	13	20	8	19	19	22	6
D10	D6_t2	3	■	■						■		ST88 (CC23)		■						6	6	9	15	18	22	30	22	32	13	17	11	21	20	6	6
D17	D6_t3	8	■	■						■		ST58 (CC155)		■					■	6	6	16	19	22	22	26	6	32	13	18	6	6	18	22	20
D18	D5_t3	8	■	■						■		ST88 (CC23)		■						6	6	14	19	19	8	25	22	33	15	24	10	21	19	22	6
D26	D19_t2	10	■	■						■		ST1722		■						6	6	12	19	20	26	30	28	29	16	22	15	20	19	20	22
D32*		15	■	■							■	ST307			■			■	■	6	6	8	14	12	6	6	6	8	13	16	6	13	8	21	6
D40		17	■	■						■		ST88 (CC23)		■						6	6	17	16	21	24	30	24	30	15	20	11	19	18	6	6
D46	D46_t1	22	■	■							■	ST307			■			■	■	6	6	14	15	16	6	6	6	8	13	17	6	13	8	22	6
D48	D48_t1	22	■	■						■		ST224	■							6	6	13	18	18	6	6	6	28	14	21	6	6	6	21	6
C15		22	■		■						■	ST307			■			■	■	6	6	8	14	14	6	6	6	7	13	16	6	12	7	21	6
C16 SK1		22	■		■					■		ST683 (CC155)				■				6	6	11	17	22	17	19	16	30	15	22	7	20	20	6	6
C16 SK2		22	■		■					■		ST69 (CC69)			■				■	6	6	10	17	18	21	21	23	30	18	20	6	20	19	22	7
C17*		22	■		■					■		ST224	■							6	6	12	17	16	6	6	6	30	14	23	6	6	6	23	6
s7*		22	■			■				■		ST224	■							6	6	14	18	18	6	6	6	30	14	23	6	6	6	23	6
j7		22	■			■					■	ST307			■			■	■	6	6	8	14	14	6	6	6	8	13	16	6	13	8	20	6
i7*		22	■			■					■	ST307			■			■	■	6	6	8	14	15	6	6	6	7	13	16	7	12	8	20	6
t7		22	■			■					■	ST307			■			■	■	6	6	8	13	12	6	6	6	7	13	16	6	11	8	21	6
w7		22	■			■					■	ST307			■			■	■	6	6	9	15	15	6	6	6	7	13	17	6	14	7	22	6
f7		22	■			■					■	ST307			■			■	■	6	6	8	15	16	6	6	6	7	12	18	6	9	7	22	6
l7		22	■			■					■	ST307			■			■	■	6	6	8	13	15	6	6	6	6	12	16	6	12	8	22	6
D54	D47_t2	24	■	■							■	ST307			■			■	■	6	6	8	14	13	6	6	6	7	12	16	7	13	7	23	6
D55	D46_t2	24	■	■							■	ST307			■			■	■	6	6	8	15	15	6	6	6	8	12	17	6	12	7	20	6
C21		24	■		■						■	ST307			■			■	■	6	6	8	14	14	6	6	6	8	13	16	6	13	8	21	6
D58 SK2*		29	■	■						■		ST38 (CC38)		■						6	6	12	17	18	6	6	6	30	8	20	8	18	15	20	19
C23*	C20_t2	29	■		■						■	ST307			■			■	■	6	6	8	14	14	6	6	6	7	12	15	6	11	7	19	6
D68		43	■	■						■		ST5204			■				■	6	7	14	18	23	20	20	6	16	15	23	6	19	19	21	6
D69 SK2		43	■	■						■		ST224	■							6	6	13	18	18	6	6	6	28	14	20	6	17	6	21	6
D69 SK3		43	■	■							■	ST15					■	■	■	6	6	8	10	15	6	6	6	20	8	15	12	6	15	16	16
i11		43	■			■				■		ST224	■							6	6	14	19	18	6	6	6	28	15	24	6	17	6	22	6
D72*		45	■	■						■		ST38 (CC38)		■						6	6	13	19	18	6	6	6	22	8	20	7	17	17	22	20
t12*		45	■			■					■	ST307			■			■	■	6	6	8	16	15	6	12	6	6	11	13	6	17	16	19	15
y12		45	■			■					■	ST307			■			■	■	6	6	8	17	16	6	13	6	6	12	14	6	20	17	20	16
H1O1_t1 SK1		27					■		■	■		ST10 (CC10)			■					6	7	14	20	22	22	26	6	31	8	26	7	23	20	27	8
H1O1_t1 SK2		27					■		■	■		ST224	■							6	6	14	17	19	6	6	6	29	16	21	6	6	6	21	7
H1CD1_t1 SK1	D48_t2	27		■			■			■		ST224	■							6	6	15	18	18	6	6	6	29	15	20	6	6	6	21	7
H1CD1_t1 SK2	D48_t2	27		■			■			■		ST5869				■				6	6	15	18	19	6	6	6	29	14	23	15	21	13	6	8
H1c1		47				■	■			■		ST224	■							6	6	12	16	17	6	6	6	29	16	21	6	6	6	21	7
H1f1		47				■	■			■		ST224	■							6	6	15	16	16	6	6	6	30	16	20	6	6	6	21	7
H1g1		47				■	■			■		ST224	■							6	6	14	6	19	6	6	6	30	16	21	6	6	6	21	7
H1h1		47				■	■			■		ST224	■							6	6	14	16	18	6	6	6	27	16	20	6	6	6	20	6
H1m1		47				■	■			■		ST224	■							6	6	13	18	16	6	6	6	26	16	21	6	6	6	23	6
H1O1_t2*		47					■		■	■		ST224	■							6	6	12	18	18	6	6	6	23	15	21	6	6	6	22	6
H1CD1_t2*	D48_t3	47		■			■			■		ST224	■							6	6	11	14	17	6	6	6	28	14	20	6	6	6	21	6
H1y1*		47				■	■			■		ST224	■							6	6	14	16	19	6	6	6	29	17	19	6	6	6	23	6
H1CD1_t3	D48_t4	57		■			■			■		ST224	■							6	6	14	16	18	6	6	6	29	16	20	6	6	6	22	6
H1CD1_t4 SK2	D48_t5	77		■			■			■		ST224	■							6	6	14	15	18	6	6	6	28	17	22	6	6	6	23	6
H1O1_t4		77					■		■	■		ST10 (CC10)			■					6	8	20	22	24	20	26	15	30	8	26	14	23	20	28	22
H2O2_t1*		68						■	■	■		ST38 (CC38)		■						6	6	15	14	19	6	6	6	30	7	20	7	20	19	22	18
H2O2_t2		118						■	■	■		ST38 (CC38)		■						6	6	17	15	22	6	6	6	30	8	23	9	20	22	22	18

AM, ampicillin; CZ, cephazolin; CTX, cefotaxime; AMC, amoxicillin-clavulanic acid; FEP, cephepim; NA, nalidixic acid; CIP, ciprofloxacin; SXT, sulfamethoxazole trimethoprim; FOS, Fosfomycin; AZM, Azithromycin; F/M, Nitrofurantoin; S, streptomycin; KM, kanamycin; GM, gentamicin; C, chloramphenicol; TE, tetracycline; *, whole genome sequencing carried out

Table S2: Questionnaire for the owners of colonized pets

Category	Question		Answer options	Household 1	Household 2
General questions	1	Please specify your gender	■ Male ■ Female	■ Female	■ Male<br>■ Female
	2	Please specify your age		41	39 (owner 1), 48 (owner 2)
	3	How many people live in your household?	■ 1 ■ 2 ■ 3 ■ 4 ■ 5 ■ 6 ■ 7 ■ 8 ■ 9 ■ 10+	■ 1	■ 2
	4	How many children live in your household?	■ 0 ■ 1 ■ 2 ■ 3 ■ 4 ■ 5 ■ 6 ■ 7 ■ 8 ■ 9 ■ 10+	■ 0	■ 0
	5	How many pets live in your household?	Dogs: ■ 0 ■ 1 ■ 2 ■ 3 ■ 4 ■ 5 ■ 6 ■ 7 ■ 8 ■ 9 ■ 10+<br>Cats: ■0 ■ 1 ■ 2 ■ 3 ■ 4 ■ 5 ■ 6 ■ 7 ■ 8 ■ 9 ■ 10+	Dogs: ■ 1<br>Cats: ■ 0<br>Others: ■ 0	Dogs: ■ 2<br>Cats: ■ 2<br>Others: ■ 0

			Others: ■0 ■ 1 ■ 2 ■ 3 ■ 4 ■ 5 ■ 6 ■ 7 ■ 8 ■ 9 ■ 10+		
	6	Where do you live?	■ City ■ Town ■ Countryside	■ Town	■ Countryside
	7	What is your job activity?		Surgical cosmetician	Care professional (works in nursing home)
	8	Do you work in the healthcare system?	■ Yes ■ No	■ No	■ Yes
	9	Do you work with animals?	■ Yes ■ No [skip Q10/11]	■ No	■ No
	10	If YES: Which animals do you work with?	■ Cattle ■ Sheep ■ Goat ■ Pig ■ Poultry ■ Horse ■ Dogs ■ Cats ■ Reptiles ■ Small rodents ■ Other	n.a.	n.a.
	11	How often do you have contact to these animals?	■ Every day ■ Every other day ■ Twice a week ■ Once a week ■ Once every two weeks ■ Once a	n.a.	n.a.

			month ■ Less often than once a month		
Hand-Washing behavior	12	I washed my hands with soap ___ times yesterday.	■ 0 ■ 1–2 ■ 3–4 ■ 5–6 ■ 7–12 ■ >12	■ >12	■ >12
	13	I have heard of antibacterial soap.	■ Yes ■ No [skip Q14/ Q15]	■ Yes	■ Yes
	14	I have antibacterial soap in my house.	■ Always ■ Often ■ Sometimes ■ Rarely ■ Never ■ I don´t know	■ Often	■ Sometimes
	15	Please select one answer	■ I sometimes prefer to use an antibacterial soap over regular soap when washing my hands. ■ I always prefer to use an antibacterial soap over regular soap when washing my hands. ■ I don't think about the benefits an antibacterial soap might have over	■ I sometimes prefer to use an antibacterial soap over regular soap when washing my hands.	■ I sometimes prefer to use an antibacterial soap over regular soap when washing my hands.

			regular soap when washing my hands. ■ I think about the benefits of an antibacterial soap but I choose not to use one		
	16	I have heard of hand sanitizer.	■ Yes ■ No [skip Q17–Q19]	■ Yes	■ Yes
	17	I have hand sanitizer in my house.	■ Always ■ Often ■ Sometimes, ■ Rarely ■ Never ■ I don´t know	■ Often	■ Always
	18	I often use a hand sanitizer when soap and water are not available.	■ Strongly agree ■ Agree ■ Neutral ■ Disagree ■ Strongly disagree	■ Disagree	■ Neutral
	19	I regularly use hand sanitizer (multiple options possible):	■ after using the toilet ■food before preparing food ■ before eating food ■ after petting my dog/ cat ■ after preparing my dog´s/	■after disposal of my pet's feces	■after disposal of my pet's feces

			cat´s food ■ after handling my dog´s/ cat´s food bowl ■after disposal of my pet's feces		
	20	I wash my hands with soap after using the toilet.	■ Always ■ Often ■ Sometimes ■ Rarely ■ Never	■ Always	■ Often
	21	I wash my hands with soap before preparing food.	■ Always ■ Often ■ Sometimes ■ Rarely ■ Never	■ Always	■ Often
	22	I wash my hands with soap before eating food.	■ Always ■ Often ■ Sometimes, ■ Rarely ■ Never	■ Always	■ Often
	23	I wash my hands with soap after petting my dog/ cat.	■ Always ■ Often ■ Sometimes ■ Rarely ■ Never	■ Rarely	■ Rarely
	24	I wash my hands with soap after preparing my dog´s/ cat´s food.	■ Always ■ Often ■ Sometimes ■ Rarely ■ Never	■ Often	■ Sometimes
	25	I wash my hands with soap after handling my dog´s/ cat´s food bowl.	■ Always ■ Often ■ Sometimes ■ Rarely ■ Never	■ Rarely	■ Rarely

	26	I wash my hands with soap after disposal of my pet's feces.	■ Always ■ Often ■ Sometimes ■ Rarely ■ Never	■ Always	■ Often
Cleaning behavior:	27	I try to make sure I do a bit of cleaning around the house	■ Every day ■ More than once a week ■ Once a week ■ Every two weeks ■ Once a month ■ Less often than once a month	■ More than once a week	■ More than once a week
	28	I wipe the floor of my house	■ Every day ■ More than once a week ■ Once a week ■ Every two weeks ■ Once a month ■ Less often than once a month	■ More than once a week	■ Once a week
	29	Please indicate what cleaning product you use to wipe the floor		Vinegar cleaning product/ commercially available floor cleaner	all purpose cleaner
Bathroom	30	I try to clean the bathroom thoroughly	■ Every day ■ More than once a week ■ Once a week ■ Once every	■ More than once a week	■ Once a week

			two weeks ■ Once a month ■ Less often than once a month		
	31	I use separate cleaning materials (e.g. separate cleaning cloth) for the bathroom.	■ Strongly agree ■ Agree ■ Neutral ■ Disagree ■ Strongly disagree	■ Strongly agree	■ Agree
	32	Please indicate what cleaning product you use to clean the bathroom		commercially available bathroom cleaner	commercially available bathroom cleaner
Kitchen	33	I try to clean the kitchen thoroughly	■ Every day ■ More than once a week ■ Once a week ■ Every two weeks ■ Once a month ■ Less often than once a month	■ Every day	■ Once a week
	34	I use a surface cleaner in the kitchen.	■ Every day ■ More than once a week ■ Once a week ■ Every two weeks ■ Once a month ■ Less often than once a month ■ Never	■ Every day	■ Once a week

	35	I try to clean the fridge thoroughly	■ More than once a week ■ Once a week ■ Every two weeks ■ Once a month ■ Once every 3 months ■ Once every 6 months ■ Less often than once a year ■ Never	■ Once every 3 months	■ Once every 3 months
	36	I wash or change kitchen towels	■ Every day ■ More than once a week ■ Once a week ■ Every two weeks ■ Once a month ■ Once every 3 months	■ Once a week	■ Once a week
	37	I use disposable (kitchen) paper towels	■ Yes ■ No	■ Yes	■ Yes
	38	I wash or change kitchen sponges	■ Every day ■ More than once a week ■ Once a week ■ Every two weeks ■ Once a month ■ Once every 3 months	■ Once a week	■ Every two weeks

	39	When preparing food, I clean the work surfaces between work steps.	■ Always ■ Often ■ Sometimes ■ Rarely ■ Never	■ Always	■ Sometimes
	40	When preparing food, I clean my hands between work steps.	■ Always ■ Often ■ Sometimes ■ Rarely ■ Never	■ Always	■ Sometimes
	41	I wash food for raw consumption, such as lettuce, vegetables and fruit.	■ Always ■ Often ■ Sometimes ■ Rarely ■ Never	■ Always	■ Always
	42	I keep meat separate from other foods.	■ Always ■ Often ■ Sometimes ■ Rarely ■ Never ■ I don´t keep meat in my house	■ Often	■ Sometimes
	43	I use separate knives for meat and foods of non-animal origin.	■ Always ■ Often ■ Sometimes ■ Rarely ■ Never ■ I don´t prepare meat in my house	■ Never	■ Never
	44	I use separate chopping boards for meat and foods of non-animal origin.	■ Always ■ Often ■ Sometimes ■ Rarely ■ Never ■ I don´t prepare meat in my house	■ Never	■ Never

Dogs and cats	45	My pet lives inside my house	■ Yes ■ No	■ Yes	■ Yes
	46	I wash my pet's toys	■ Every day ■ More than once a week ■ Once a week ■ Every two weeks ■ Once a month ■ Rarely ■ Never	■ Never (toys will be thrown away and not washed)	■ Rarely
	47	I wash my pet's bed	■ Every day ■ More than once a week ■ Once a week ■ Every two weeks ■ Once a month ■ Rarely ■ Never	■ Once a month	■ Rarely
	48	I hoover my pet's bed	■ Every day ■ More than once a week ■ Once a week ■ Every two weeks ■ Once a month ■ Rarely ■ Never	■ More than once a week	■ Every two weeks
	49	I clean my pet's food bowl	■ Every day ■ More than once a week ■ Once a week ■ Every two	■ Every day	■ Every day

			weeks ■ Once a month ■ Rarely ■ Never		
	50	I clean my pet's food bowl (multiple answers possible)	■ With soap ■ With water ■ With a separate cloth ■ With the same cloth that I use for my other dishes ■ In the dishwasher	■ With soap ■ With water ■ In the dishwasher	■ With water
	51	My pet is allowed on the bed	■ Yes ■ No	■ No	■ Yes
	52	If Yes: How often does your pet stay/ sleep in the bed?	■ Every day ■ More than once a week ■ Once a week ■ Every two weeks ■ Once a month ■ Rarely ■ Never	n.a.	■ More than once a week
	53	My pet is allowed on the sofa	■ Yes ■ No	■ Yes	■ Yes
	54	If Yes: How often does your pet stay/ sleep on the sofa?	■ Every day ■ More than once a week ■ Once a week ■ Every two weeks ■ Once a month ■ Rarely ■ Never	■ Every day	■ Every day

	55	My pet is allowed to lick my hands	■ Yes ■ No	■ No	■ Yes
	56	My pet is allowed to lick my face	■ Yes ■ No	■ No	■ No
	57	I groom my pet	■ Every day ■ More than once a week ■ Once a week ■ Every two weeks ■ Once a month ■ Rarely ■ Never	■ Rarely	■ Rarely
	58	I remove my cat's feces from the litter box (only show this question for cat owners)	■ Every day ■ More than once a week ■ Once a week ■ Rarely ■ My cat doesn't use a litter box	n.a.	■ Once a week
	59	I clean (e.g. change sand, wash, disinfect) my cat's litter box (only show this question for cat owners)	■ Every day ■ More than once a week ■ Once a week ■ Rarely ■ My cat doesn't use a litter box	n.a.	■ Rarely

	60	I clean up my dog's feces from the garden (only show this question for dog owners)	■ Every day ■ More than once a week ■ Once a week ■ Every two weeks ■ Once a month ■ Rarely ■ Never ■ My dog is not allowed to defecate in the garden ■ I don´t have a garden	■ Every day	■ Every day
	61	I feed my pet the following diet (multiple answers possible):	■ Dry food [skip Q62–64] ■ Canned/ semi-moist food [skip Q62–64] ■ Home-cooked [skip Q62–64] ■ Raw food	■ Dry food ■ Canned/ semi-moist food ■ Home-cooked	■ Dry food ■ Canned/ semi-moist food ■ Home-cooked
	62	If raw food, please specify which kind (multiple answers possible):	■ Vegetable ■ Fruit ■ Chicken ■ Beef ■ Pork ■ Other:________	n.a.	n.a.
	63	If raw food, please specify where you purchase it (multiple answers possible):	■ Pet shop ■ Butcher ■ Supermarket ■ Online ■ Other:________	n.a.	n.a.

	64	If raw food, please specify how you store it (multiple answers possible):	■ Separate freezer ■ Separate fridge ■ Same freezer as I use for my own food ■ Same fridge as I use for my own food ■ Other:________	n.a.	n.a.
	65	Please indicate if one of the following applies:	■I am Pregnant ■Pregnant person in household ■I am Immunocompromised ■ Immunocompromised person in household ■Children <2 years old in household ■People >65 years old ■Does not apply	■Does not apply	■Does not apply
	66	If you wish, please feel free to specify any current illnesses	■No current illness ■_________	■No current illness	■No current illness
	67	Have you had contact with the human health care system in the past 6 months?	■Yes ■ No [skip Q68/69]	■Yes	■Yes

	68	If YES, which kind?	■ General practitioner visits ■ Hospital visits ■ Hospitalization ■ Nursing home visits ■ Relatives receiving health care ■ Relatives working in health care	■ General practitioner visits	■ General practitioner visits ■ Nursing home visits
	69	If hospitalization, how many days?		n.a.	n.a.
	70	Have you had diarrhea during the past 12 months?	■ Yes ■ No [skip Q71–73]	■ Yes	■ Yes (Norovirus)
	71	If yes, when?	■ At the moment ■ <3 months ago ■ <6 months ago ■ <12 months ago	■ <3 months ago	■ <12 months ago
	72	If yes, did you take anti-diarrheal drugs?	■ Yes ■ No	■ No	■ No
	73	If yes, do you currently take anti-diarrheal drugs?	■ Yes ■ No	■ No	■ No

	74	Have you been diagnosed with a bacterial gastrointestinal disease (e.g., Salmonella) in the last 2 years?	■ Yes ■ No [skip Q75/76]	■ No	■ No
	75	If yes, when?	■ At the moment ■ <3 months ago ■ <6 months ago ■ <1 year ago ■ <2 years ago	n.a.	n.a.
	76	If Yes: indicate which pathogen and approx. date (if you remember):		n.a.	n.a.
	77	Have you ever received antibiotic treatment in the past?	■ Yes ■ No [skip Q78/79]	■ Yes	■ Yes
	78	If yes, when?	■ At the moment ■ <3 months ago ■ <1 year ago ■ <2 years ago ■ > 2 years ago ■ > 5 years ago	■ > 5 years ago (20 years ago)	■ > 2 years ago
	79	If yes, which antibiotic?		n.k.	Amoxicillin and Clavulanic Acid

Diet	80	What kind of diet do you usually follow?	■ Omnivore ■ Vegetarian ■ Vegan ■ Rawist	■ Omnivore	■ Omnivore
	81	I eat raw meat and/or raw fish ______.	■ Every day ■ Every other day ■ Twice a week ■ Once a week ■ Every two weeks ■ Once a month ■ Rarely ■ Never	■ Rarely	■ Rarely
	82	I eat fresh vegetables and/or fruit ______.	■ Every day ■ Every other day ■ Twice a week ■ Once a week ■ Every two weeks ■ Once a month ■ Rarely ■ Never	■ Every other day	■ Twice a week
	83	I eat fresh cheese and/or dairy products ______.	■ Every day ■ More than once a week ■ Once a week ■ Every two weeks ■ Once a month ■ Rarely ■ Never	■ More than once a week	■ Every day
Travel	84	Did you visit other countries for more than two days (>2 days) during the last year?	■ Yes ■ No [skip Q85]	■ Yes	■ Yes

	85	If Yes, please select where (multiple answers possible):	■ Europe ■ Asia ■ North America ■ South America ■ Australia ■ Africa ■ Antarctica	■ Europe ■ Asia	■ Europe
	86	Please feel free to provide city/country, number of days, and when:		■ Thailand<br>■ Italy (Como Lake)<br>■ Mallorca<br>■ Germany (Allgäu)	■ Italy<br>■ Austria<br>■ Germany
Other	87	Is there anything else you would like us to know?		no	Due to work in nursing home daily contact to patients.<br>Colonized dog has diabetes.

n.a., not applicable; n.k., not known

Table S3: List of high-touch surfaces in the veterinary clinic

**Abbreviation (strain ID)**	**Surface**
a	oxygen cage
b	phone
c	PC keyboard
d	PC mouse
e	examination table
f	water tap
g	drawer
h	large cabinet
i	small cabinet
j	area of drug preparation

k	storage box
l	fridge with medication
m	infusion pump
n	thermometer
o	stethoscope
p	soap
q	alcohol
r	cat cage
s	dog cage
t	blood pressure monitor
u	wastewater channel
v	floor

w	scale (floor)
x	scale (display)
y	scissors

Table S4: List of high-touch surfaces in the households of colonized pets

**Abbreviation (strain ID)**	**Surface household 1**	**Surface household 2**
a	leash	
b	dog’s blanket (car)	dog’s sleeping basket
c	water bowl	
d	feeding bowl	
e	dog/ cat toy	
f	dog’s sleeping basket (living room)	dog’s sleeping basket
g	dog’s blanket on terrace	cat’s sleeping basket
h	dog’s sleeping basket (bedroom)	sunbed
i	drain	
j	bidet	cat’s toilet

k	toilet	
l	floor	
m	carpet	
n	kitchen drain	
o	drain (shower)	bed
p	door handle	
q	washing machine	
r	bathroom guests	carpet bathroom
s	door handle	
t	door handle (entrance)	
u	sofa	
v	dishwasher	furniture (living room)

w	Fridge
x	cleaning sponge
y	kitchen sponge
